# Melatonin Improves the Photosynthetic Apparatus in Pea Leaves Stressed by Paraquat *via* Chlorophyll Breakdown Regulation and Its Accelerated *de novo* Synthesis

**DOI:** 10.3389/fpls.2017.00878

**Published:** 2017-05-29

**Authors:** Katarzyna Szafrańska, Russel J. Reiter, Małgorzata M. Posmyk

**Affiliations:** ^1^Laboratory of Plant Ecophysiology, Faculty of Biology and Environmental Protection, University of ŁódźŁódź, Poland; ^2^Department of Cellular and Structural Biology, University of Texas Health Science Center San AntonioSan Antonio, TX, United States

**Keywords:** ALA, chlorophyll, chlorophyllase, chlorophyllide, hydropriming, melatonin, oxidative stress, pheophytin

## Abstract

The positive effect of melatonin on the function of the photosynthetic apparatus is known, but little is known about the specific mechanisms of melatonin's action in plants. The influence of melatonin on chlorophyll metabolism of 24-day-old *Pisum sativum* L. seedlings during paraquat (PQ)-induced oxidative stress was investigated in this study. Seeds were hydro-primed with water (H), 50 and 200 μM melatonin/water solutions (H-MEL50, H-MEL200), while non-primed seeds were used as controls (C). Increases in chlorophyllase activity (key enzyme in chlorophyll degradation) and 5-aminolevulinic acid contents (the first compound in the porphyrin synthesis pathway) were observed in H-MEL50 and H-MEL200 leaf disks. This suggests that melatonin may accelerate damaged chlorophyll breakdown and its *de novo* synthesis during the first hours of PQ treatment. Elevated level of pheophytin in control leaf disks following 24 h of PQ incubation probably was associated with an enhanced rate of chlorophyll degradation through formation of pheophytin as a chlorophyll derivative. This validates the hypothesis that chlorophyllide, considered for many years, as a first intermediate of chlorophyll breakdown is not. This is indicated by the almost unchanged chlorophyll to chlorophyllide ratio after 24 h of PQ treatment. However, prolonged effects of PQ-induced stress (48 h) revealed extensive discolouration of control and water-treated leaf disks, while melatonin treatment alleviated PQ-induced photobleaching. Also the ratio of chlorophyll to chlorophyllide and porphyrin contents were significantly higher in plants treated with melatonin, which may indicate that this indoleamine both retards chlorophyll breakdown and stimulates its *de novo* synthesis during extended stress. We concluded that melatonin added into the seeds enhances the ability of pea seedlings to accelerate chlorophyll breakdown and its *de novo* synthesis before stress appeared and for several hours after, while during prolonged PQ incubation melatonin delays chlorophyll degradation.

## Introduction

Melatonin (MEL) as a small amphiphilic particle can easily cross cell membranes and penetrate all cellular compartments. Due to the ability to scavenge reactive oxygen and nitrogen species (ROS and RNS), and its high diffusibility, it is considered as a powerful antioxidant with a very large range of actions (Manchester et al., 2015; Reiter et al., 2016). ROS and RNS generated under harmful environmental conditions inflict damage to critical macromolecules, such as lipids, proteins, DNA, etc.; thus MEL plays an important role in the defense system of plants (Reiter et al., 2015). A potent antioxidant function of MEL is associated not only with its ability to directly scavenge ROS and RNS or to stimulate antioxidant enzymes activities, but also due to its ability to generate a highly effective free radical scavenging cascade of its metabolites, including cyclic 3-hydroxymelatonin (C-3HOM), N^1^-acetyl-N^2^-formyl-5-methoxyknuramine (AFMK) and N-acetyl-5-methoxykynuramine (AMK) (Galano et al., 2013). Because of this cascade, MEL is considered a more effective universal antioxidant than many other substances with known and proven antioxidant properties, such as vitamin C, vitamin E, glutathione, and NADH (Tan et al., 2015). The antioxidant cascade provided by MEL and its metabolites makes this indoleamine, even at low concentrations, highly effective in protecting organisms against oxidative stress (Galano et al., 2013).

Although a multitude of physiological, biochemical, and molecular processes determine plant growth and development, photosynthesis is a key factor. It is the most fundamental and intricate physiological process in all green plants which is severely affected by environmental stresses (Ashraf and Harris, 2013). Degradation and loss of chlorophyll (Chl) is one of the biochemical markers of aging but a positive effect of MEL on this process has been reported. According to Wang et al. (2012, 2013a,b), MEL delays drought- and dark-induced leaf senescence in apple due to maintaining the photosystem II (PSII) function under stress and reducing the typical decline in Chl content. Moreover, in leaves treated with MEL the expression of a key genes for the Chl degradation - pheide *a* oxygenase (PAO), senescence-associated gene 12 (SAG12) and sugar-sensing and senescence associated hexokinase-1 gene (HXK1)- are also inhibited. In *Arabidopsis thaliana* leaves treated with paraquat (PQ), MEL significantly decreased the expression of chlorophyllase (CLH1) gene that is a light-regulated enzyme participating in Chl degradation (Weeda et al., 2014). In these leaves exogenous MEL clearly slowed Chl loss, as also seen in barley leaves during senescence (Arnao and Hernandez–Ruiz, 2009) and in rice leaves under salt stress (Liang et al., 2015). Hence, due to its ability to support photosynthesis, MEL plays an important role in senescence delay.

**Figure 1:**
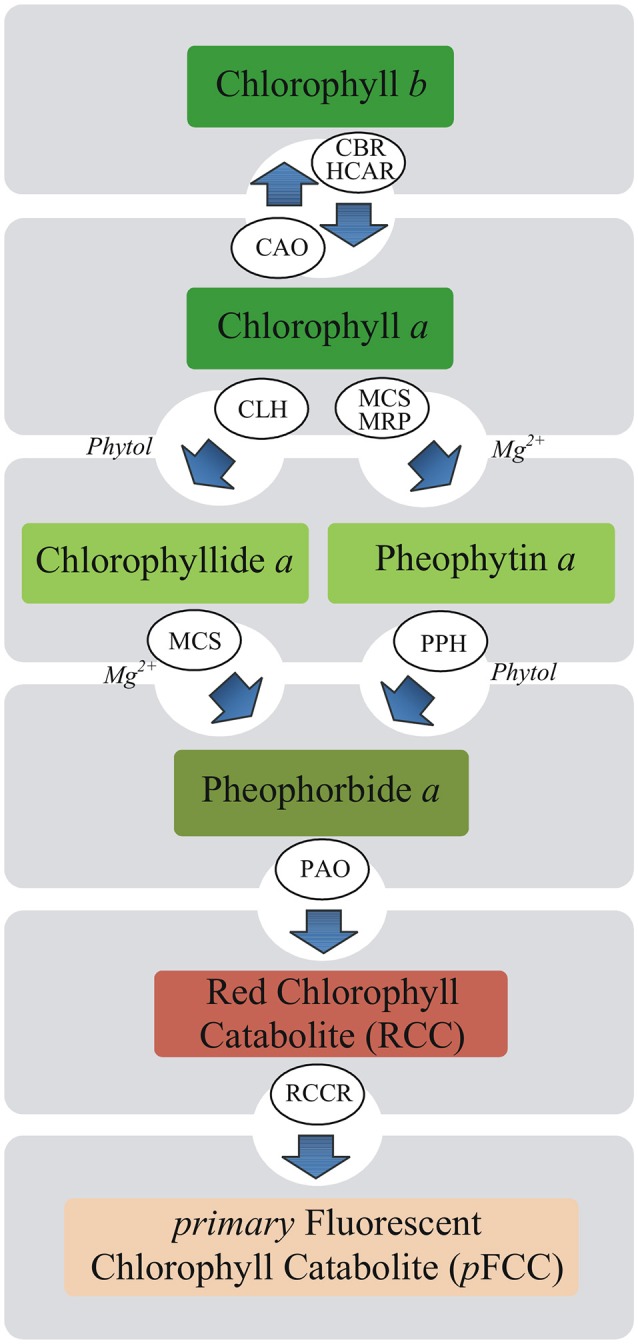
Putative pathways of chlorophyll degradation in plants. Enzymes involved in these processes are as follows: CAO, chlorophyll *a* oxygenase; CBR, chlorophyll *b* reductase; CLH, chlorophyllase (Chlase); HCAR, 7-hydroxymethyl chlorophyll *a* reductase; MCS, metal-chelating substance; MRP, metal-releasing protein; PAO, pheophorbide *a* oxygenase; PPH, pheophytinase; RCCR, red chlorophyll catabolite reductase.

The mechanism whereby MEL influences photosynthesis remains unknown. In plants under stressful conditions, maintaining a balance between the breakdown of damaged Chl and its *de novo* synthesis is important. Rapid degradation of free Chl or its colored derivatives is necessary to avoid cell damage due to their photodynamic action (Takamiya et al., 2000). Chl breakdown is also a direct precondition for the remobilization of proteins, chloroplast lipids, and metals (Christ and Hörtensteiner, 2014). Therefore, this process takes place not only under stressful conditions but also during various phases of the life cycle of plants. Chl breakdown is a multistep enzymatic process and Chl degradation into phytol, and the first decomposition product of the porphyrin ring, takes place in four successive steps catalyzed by chlorophyllase (Chlase), Mg-dechelatase, PAO and red chlorophyll catabolite reductase (Harpaz-Saad et al., 2007; Figure 1). Chlase catalyzes the reaction of Chl hydrolysis to chlorophyllide (Chlide) and phytol and it is the first enzyme of Chl catabolism during fruit ripening and leaf senescence (Takamiya et al., 2000; Hörtensteiner, 2006). However, in some plants Chlase was found not to be essential for dephytylation. Recent studies have shown that the chlorophyll degradation may progress through the formation of pheophytin *a* (Pheo *a*) as a chlorophyll derivative (Schelbert et al., 2009; Dissanayake et al., 2012). Based on these findings, it is assumed that Chl degradation pathway is not necessarily *via* Chlide *a* but also involves Pheo *a*.

**Figure 2:**
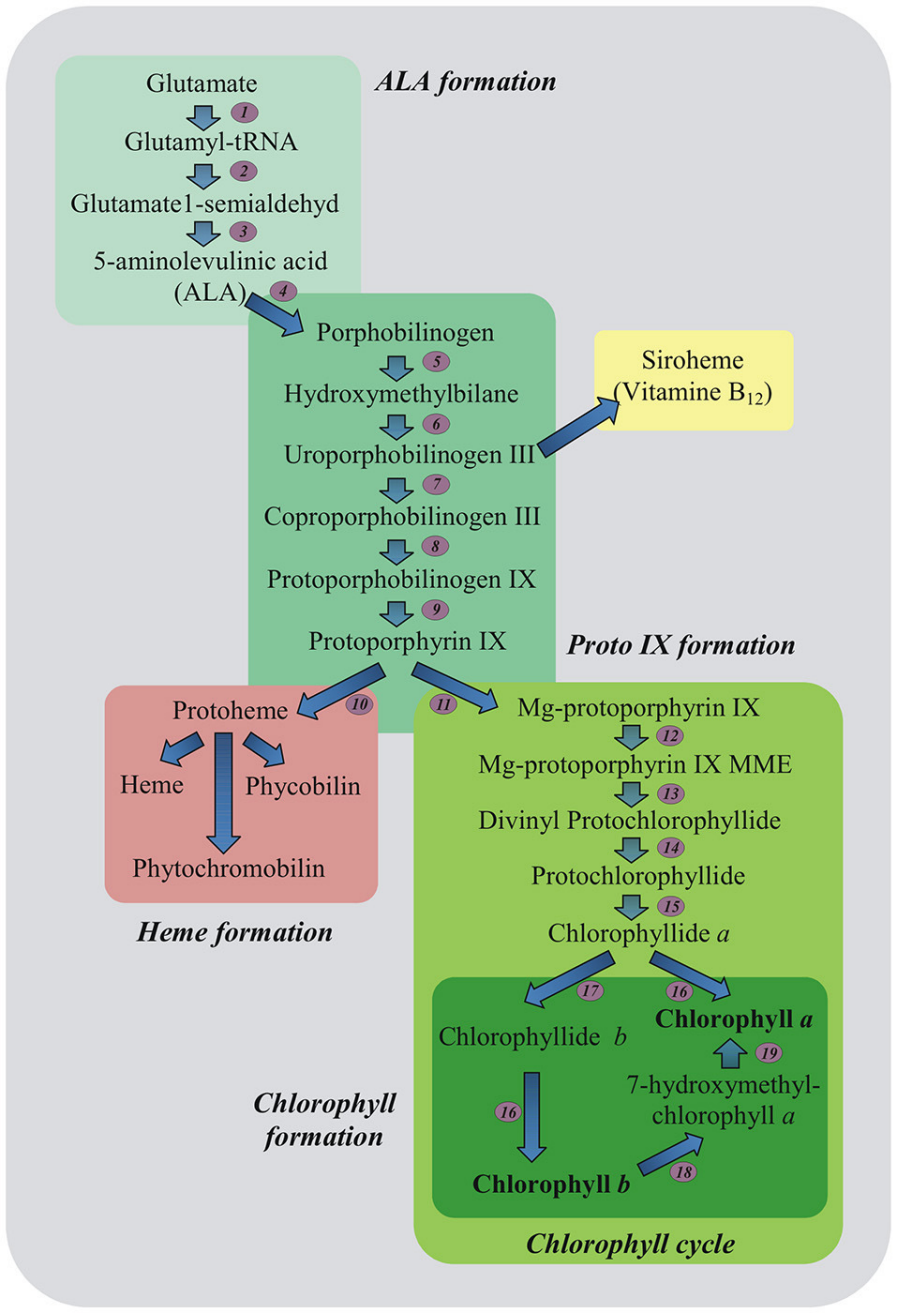
Plant tetrapyrrole biosynthesis pathway. 5-aminolevulinic acid (ALA, the universal precursor of all tetrapyrroles) is synthesized from glutamate. ALA is then converted into protoporphyrin IX before the pathway branches into haem and chlorophyll biosynthesis. The ratio of chlorophyll *a* and *b* is balanced in the chlorophyll cycle. Biosynthetic enzymes: *1*-GluRS, glutamyl-tRNA synthetase; *2*-GluTR, glutamyl-tRNA reductase; *3*-GSAT, glutamate-1-semialdehyde aminotransferase; *4*-ALAD, ALA dehydratase; *5*-HB synthase, hydroxymethylbilane synthase; *6*-Uro III synthase, uroporphyrinogen III synthase; *7*-Uro III decarboxylase, uroporphyrinogen III decarboxylase; *8*-Copro III oxidase, coproporphyrinogen III oxidase; *9*-PPOX, protoporphyrinogen IX oxidase; *10*-FeCh, Fe chelatase; *11*-Mg dechelatase; *12*-MTF, Mg protoporphyrin IX methyltransferase; *13*-cyclase; *14*-DVR, divinyl protochlorophyllide reductase; *15*-POR, light-dependent NADPH-protochlorophyllide oxidoreductase; *16*-Chl synthase, chlorophyll synthase; *17*-CAO, chlorophyll *a* oxygenase; *18*-CBR, chlorophyll *b* reductase; *19*-HCAR, 7-hydroxymethyl chlorophyll *a* reductase; MME, monomethylester (Czarnecki and Grimm, 2012, modified).

A marked decline in content of important photosynthetic pigments also occurs due to stress-induced impairment in pigment biosynthetic pathways (Ashraf and Harris, 2013; Figure 2). Down-regulation of Chl biosynthesis may be attributed to reduced accumulation of 5-aminolevulinic acid (ALA) being the precursor of all tetrapyrroles and protochlorophyllide (Santos, 2004), as well as to decreased activities of Chl biosynthetic pathway enzymes including: ALA dehydratase (4), hydroxymethylbilane synthase (5), coproporphyrinogen III oxidase (8), protoporphyrinogen IX oxidase (9), Mg-protoporphyrin IX methyltransferase (12) and protochlorophyllide oxidoreductase (15) (Turan and Tripathy, 2015; Figure 2). Since the regulation of Chl biosynthesis and degradation under stress conditions is important, it is essential to check what role in these processes may relate to MEL.

Much of the research on the role of MEL in plants is based on watering or spraying them with MEL solution. In present work, the method of MEL application was seed priming, which is a well-known technique for seed quality improvement (Jisha et al., 2013). To determine the role of MEL in Chl protection, pea leaves were subjected to oxidative stress. PQ was used as a stress agent; this is a fast-acting, non-selective herbicide that works in the chloroplast by diverting electrons from photosystem I (PSI). By accepting a single electron, PQ generates a stable reduced cation radical rapidly reacting with dioxygen to generate superoxide (${\text{O}}_{2}^{•-}$) (Hawkes, 2014). This in turn produces a variety of ROS, including hydrogen peroxide (H_2_O_2_) and hydroxyl radicals (^•^OH); the latter is an especially highly reactive agent that induces protein and pigment degradation, lipid peroxidation and nucleic acid damage. By affecting key components of plant cell metabolism these changes eventually cause cell death (Lascano et al., 2012).

The aim of this study was to verify whether the positive effect of MEL in PQ-treated pea leaves is associated only with limited degradation of Chl, or perhaps with increased *de novo* synthesis as well.

## Materials and methods

### Plant material

*Pisum sativum* L. seeds obtained from TORSEED (Torun, Poland) were hydro-primed with water (H) or with 50 and 200 μM melatonin/water solutions (H-MEL50, H-MEL200), while non-primed seeds were used as a controls (C) (Posmyk et al., 2008; Szafrańska et al., 2016). Melatonin concentrations were chosen as optimal, on the basis of previous experiments (Szafrańska et al., 2014). A fungicide, Thiuram (Organica-Sarzyna, Poland), was used for seed sterilization, and thereafter they were placed in plastic boxes with cottonwool moistened with distilled water and germinated at 25°C for 3 days. The young seedlings were transplanted into plastic pots filled with sterilized universal soil and pearlite (3:1); they were grown for 21 days in a breeding room at constant temperature of 25°C and a fixed photoperiod (16 h light/8 h dark) with intensity of light: 770–840 μmol m^−2^ s^−1^.

### Stress conditions

Paraquat (PQ, methyl viologen, 1,1′-dimethyl 4,4′-bipyridinium dichloride), purchased in Sigma-Aldrich (Germany), was used to generate oxidative stress in tissues. Leaf disks 18 mm in diameter were cut from 24-day-old pea plants. One part of the leaf disks was immediately used for analysis (T_0_), and the other was transferred into Petri dishes filled with 15 mL of 75 μM PQ. They were incubated in a growth chamber (Orbis DATA LOG) with constant light (350 – 370 μmol m^−2^ s^−1^), at 25°C for specified time for different analysis: 1, 2, 4, and 6 h for Chlase activity and ALA content, and 24, 48 h for Chl, carotenoid (Car), Pheo, Chlide, and porphyrin contents. After the times indicated, leaf disks were removed from the Petri dishes, dried on a paper towels, frozen in liquid nitrogen and stored at −80°C for further analysis. Only Chlase activity and ALA content were analyzed in the fresh tissues.

### Chlorophyllase (Chlase) activity

Enzyme extraction and assay were performed according to Gupta et al. (2012). The leaf disks (0.8 g) were homogenized in 5 mL of cold extraction buffer (100 mM potassium phosphate (pH 7.0), 50 mM potassium chloride, 5 mM sodium diethyldithiocarbamate (DECA), 1 mM diethylenetriaminepentaacetic acid (DPTA), 0.24% (v/v) Triton X-100) and 0.2 g pre-swollen insoluble polyvinyl pyrrolidone (PVP). The homogenate was centrifuged at 15,000 × *g* for 15 min and the supernatant was used as a crude enzyme. All these steps were performed on ice.

Chlase activity was determined in 2 mL reaction mixture containing 80 mM phosphate buffer (pH 7.0), 0.24% (v/v) Triton X-100, 0.22 μmol Chl *a* dissolved in acetone and 1 mL of crude enzyme. The reaction was run for 15 min in dark at 40°C. Aliquots of 0.5 ml were added to 5 mL of phase separation mixture containing acetone/hexane/10 mM KOH (2:3:0.2, v/v) and mixed by vortexing for 30–40 s. The resulting emulsion was centrifuged at 12,000 × *g* for 5 min for quick phase separation. Chlide *a* was estimated in acetone phase at 667 nm (spectrophotometer Hitachi U-2001) and its amount was calculated using extinction coefficient 74.9 mM^−1^ cm^−1^. One unit of activity was defined as the amount of enzyme hydrolyzing 1 nmol Chl *a* per s at 40°C. Protein in the enzyme extract was assayed according to Bradford (1976) using bovine serum albumin as a standard. Chlase activity was measured in 9 replicates (*n* = 9).

### 5-aminolevulinic acid (ALA) content

5-Aminolevulinic acid (ALA) contents were estimated according to modified method of Turan and Tripathy (2015). The leaf disks (0.2 g) were collected at different time points (0, 1, 2, 4, and 6 h of PQ treatment). They were homogenized in a cooled mortar and pestle in 2 mL of 1 M sodium acetate buffer (pH 4.6). Next homogenate was centrifuged at 10,000 × *g* for 10 min and obtained supernatant was used for analysis. The reaction mixture consisting of: 4 mL of distilled water, 1 mL of supernatant, and 250 μL of acetyl-acetone was mixed and boiled for 10 min. Next it was cooled and an equal volume of Ehlrich reagent (2 g DMBA, 30 mL of glacial acetic acid, 16 mL of 70% perchloric acid filled up to 50 mL with glacial acetic acid) was added and mixed. After 20 min of incubation absorbance at 555 nm was measured (spectrophotometer Hitachi U-2001). ALA content was evaluated from the calibration curve based on known concentrations of ALA. All assays were performed in 9 replicates (*n* = 9).

### Chlorophyll *a*+*b*: carotenoids ratio

Chls *a*+*b* and Cars were quantified spectrophotometrically according to Lichtenthaler and Buschmann (2001). The leaf disks (0.025 g) were homogenized in a cooled mortar and pestle in 5 mL of 80% acetone with MgCO_3_ and filtered. In the obtained supernatant absorbance at 470, 646, and 663 nm (spectrophotometer Hitachi U-2001) was measured. Chls *a, b, a*+*b* and Car concentrations were calculated with the following formulas:

Chl *a* [μg mL^−1^] = 12.25 × A663 − 2.79 × A646,
Chl *b* [μg mL^−1^] = 21.50 × A646 − 5.10 × A663,
Chls *a* + *b* [μg mL^−1^] = 7.15 × A663 + 18.71 × A646,
Cars [μg mL^−1^] = (1,000 × A470 − 1.82 Chl *a* − 85.02 Chl *b*)/198.
Pigment assays were performed in at least 5 replicates (*n* = 5).

### Chlorophyll: pheophytin ratio

Chl and Pheo contents were assayed by the modified method of Radojevič and Bashkin (2006). The leaf disks (0.025 g) were homogenized in a cooled mortar and pestle in 5 mL of 90% acetone with 1% of magnesium carbonate and filtered through filter paper. Aliquots of 3 mL of final extracts were placed into 1 cm cuvettes and absorbance at 664 and 750 nm was measured (A_664a_, A_750a_) (spectrophotometer Hitachi U-2001). Then 0.1 mL of 0.1 M HCl was added, mixed and absorbance at 665 and 750 nm was measured (A_665b_, A_750b_). Chl *a* and Pheo *a* concentrations were calculated from formulas:

Chl *a* [mg g^−1^ FW] = [((A_664a_ − A_750a_) − (A_665b_ − A_750b_)) × V_1_]/ 26.7 × d × E × V_2_,
Pheo *a* [mg g^−1^ FW] = [((1.7 × (A_665b_ − A_750b_) − (A_664a_ − A_750a_)) × V_1_]/ 26.7 × d × E × V_2_.

The value 26.7 above in the equations is the absorbance correction and equal A × K where A is the absorbance coefficient for chlorophyll *a* at 664 nm (11.0) and K is a ratio expressing the correction for acidification (2.43 = 1.7 × 0.7) (APHA et al., 2005). If the ratio of A_664_ before acidification and A_665_ after acidification (A_664_/A_665_) is 1.7, the sample is considered to be free of Pheo *a*. Explanation of abbreviations: d–optical distance [cm]; E–equivalent of fresh weight [g FW mL^−1^]; V_1_–sample volume [L], V_2_–extract volume in a sample [mL]. These assays were performed in 5 replications (*n* = 5).

### Chlorophyll: chlorophyllide ratio

Chl and Chlide contents were determined according to modified method of Harpaz-Saad et al. (2007). The leaf disks (0.2 g) were homogenized in a prechilled mortar and pestle in 6 mL of 100% acetone and filtered through filter paper. Aliquots of 2 mL from each sample were added to a centrifuge tubes containing 3 mL of hexane and 0.5 mL of 10 mM KOH, shortly vortexed and centrifuged for phase separation at 12,000 × *g* for 2 min. Chl levels were measured in the hexane phase, and Chlide levels were measured in the acetone phase spectrophotometrically (Hitachi U-2001) using formulas:

Chl *a* (Chlide *a*) [μg mL^−1^] = 12.7 × A_663_ − 2.69 × A_645_ and,
Chl *b* (Chlide *b*) [μg mL^−1^] = 22.9 × A_645_ − 4.68 × A_663_, as reported Arnon (1949).
Experiment was performed in 3 replicates (*n* = 3).

### Porphyrin content

Porphyrin content was estimated by method described by Sarropoulou et al. (2012). The leaf disks (0.1 g) were placed in glass test tubes and 15 mL of 96% (v/v) ethanol was added. The samples were incubated in a water bath at temperature of 65°C until total discolouration of samples (3 h). Protoporphyrin (Proto), Mg-protoporphyrin (MgProto) and protochlorophyllide (Pchlide) concentrations were calculated using the following three equations:

Proto [μg g ^−1^ FW] = [(12.25 × A_665_–2.55 × A_649_) × volume of supernatant (mL)/sample weight (g)]/892,
MgProto [μg g ^−1^ FW] = [(20.31 × A_649_–4.91 × A_665_) × volume of supernatant (mL)/sample weight (g)]/906,
Pchlide [mg g ^−1^ FW] = [(196.25 × A_575_–46.6 × A_590_–58.68 × A_628_) + (61.81 × A_590_–23.77 × A_575_–3.55 × A_628_) + (42.59 × A_628_–34.32 × A_575_–7.25 × A_590_)] × volume of supernatant (mL)/sample weight (g) × 1,000.
Porphyrin determinations were performed in at least 8 replicates (*n* = 8).

### Statistical analyses

The results represent the average values ± standard error (±SEM) of the mean. The data were analyzed using STATISTICA v.10.0_MR1_PL [StatSoft] software. The two-way or one-way (the latter applies only to **Figures 8A–C** data) analysis of variance (ANOVA) and then the *post-hoc* Duncan multiple range test were performed to find the significant differences at least *p* < 0.001 in each experiment.

## Results

**Figure 3:**
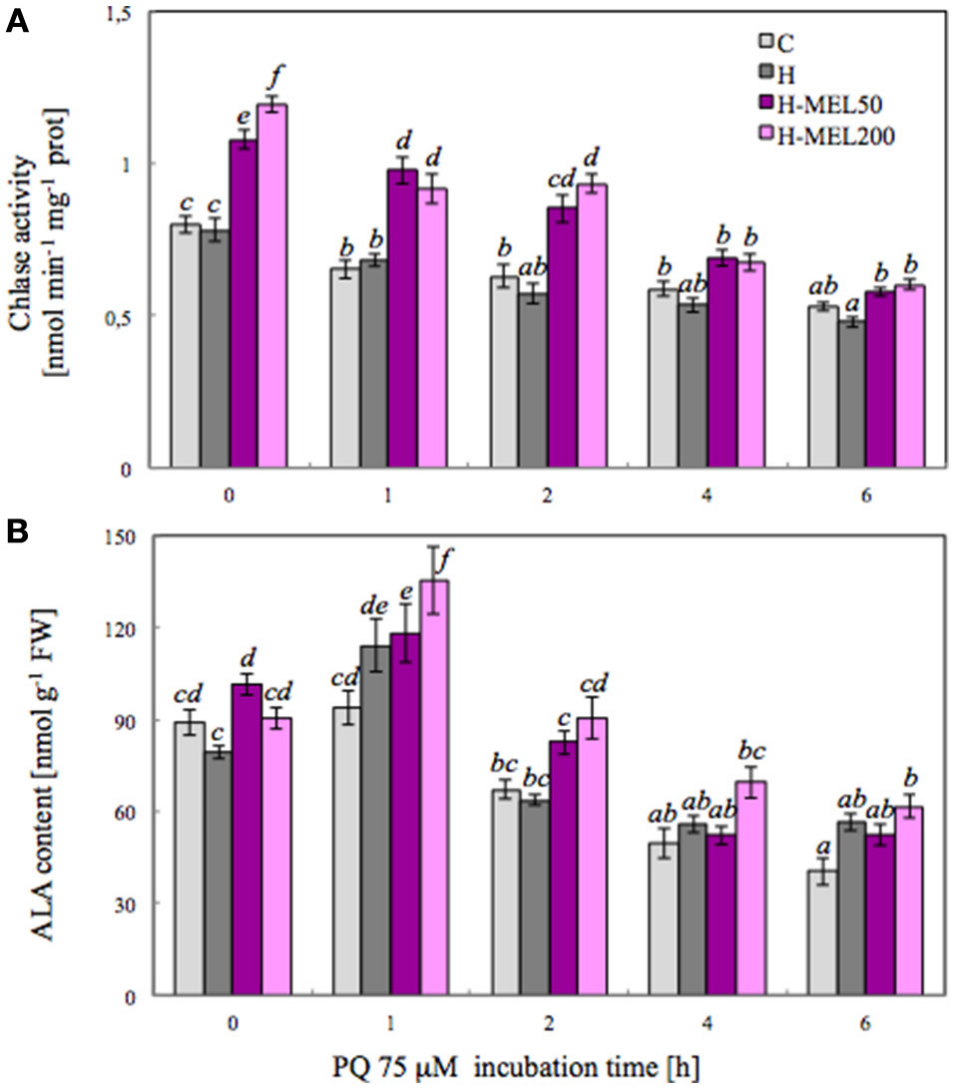
Chlorophyllase (Chlase) activity **(A)** and 5-aminolevulinic acid (ALA) content **(B)** in leaf disks cut from 24-day-old pea plants grown from the control (C), hydroprimed (H), and hydroprimed with an aqueous solution of 50 μM melatonin (H-MEL50) or 200 μM melatonin (H-MEL200) seeds. Measurements were performed at T_0_ (before PQ treatment) and after 1, 2, 4, and 6 h of disks incubation in 75 μM PQ solution. The results are expressed as mean values of about 9 measurements ± SEM. Two-way ANOVA and Duncan's *post-hoc* test were performed. The small letters on the graphs show statistical significance. ANOVA results: Chlase activity-Variant *F*_(3; 157)_ = 89.6 *p* < 0.0001; Time (T_0_, 1, 2, 4, and 6 h) *F*_(4; 157)_ = 108 *p* < 0.0001; and interaction Variant × Time *F*_(12; 157)_ = 5.7 *p* < 0.0001; ALA-Variant *F*_(3; 181)_ = 16.1 *p* < 0.0001; Time (T_0_, 1, 2, 4, and 6 h) *F*_(4; 181)_ = 87.6 *p* < 0.0001; and interaction Variant × Time *F*_(12; 181)_ = 2.4 *p* < 0.001.

To investigate the influence of exogenous MEL on Chl metabolism under PQ-induced oxidative stress, the activity of Chlase, a key enzyme involved in the chlorophyll breakdown, was analyzed. Activity of this enzyme measured after PQ treatment gradually decreased in all examined variants (Figure 3A). There were no differences between control and H leaf disks, but in variants with MEL Chlase activity was significantly higher. Before PQ treatment (T_0_), Chlase activity in MEL50 and MEL200 leaf disks was accounted for 135 and 150% of activity in control disks, respectively. This marked difference between non-treated (control and H) disks and those treated with MEL (H-MEL50, H-MEL200) variants remained at a similar level after 1 and 2 h of PQ treatment, but after 4 and 6 h the differences were reduced (Figure 3A).

5-Aminolevulinic acid (ALA) is a key precursor in the biosynthesis of porphyrin, such as Chl, so we analyzed its content. At T_0_ time point the highest ALA content was detected in H-MEL50 leaf disks (Figure 3B). PQ triggered a rapid, significant increase after 1 h of treatment and the maximum was noticed in H-MEL200 leaf disks. In H and H-MEL50 leaf disks, this level was slightly reduced, but in control variants it was lower by about 30%, compared with H-MEL200. The next 2, 4, and 6 h of PQ incubation resulted in gradual reduction of ALA content, but still the lowest content was detected in control disks and the highest in H-MEL200 leaf disks (Figure 3B).

**Figure 4:**
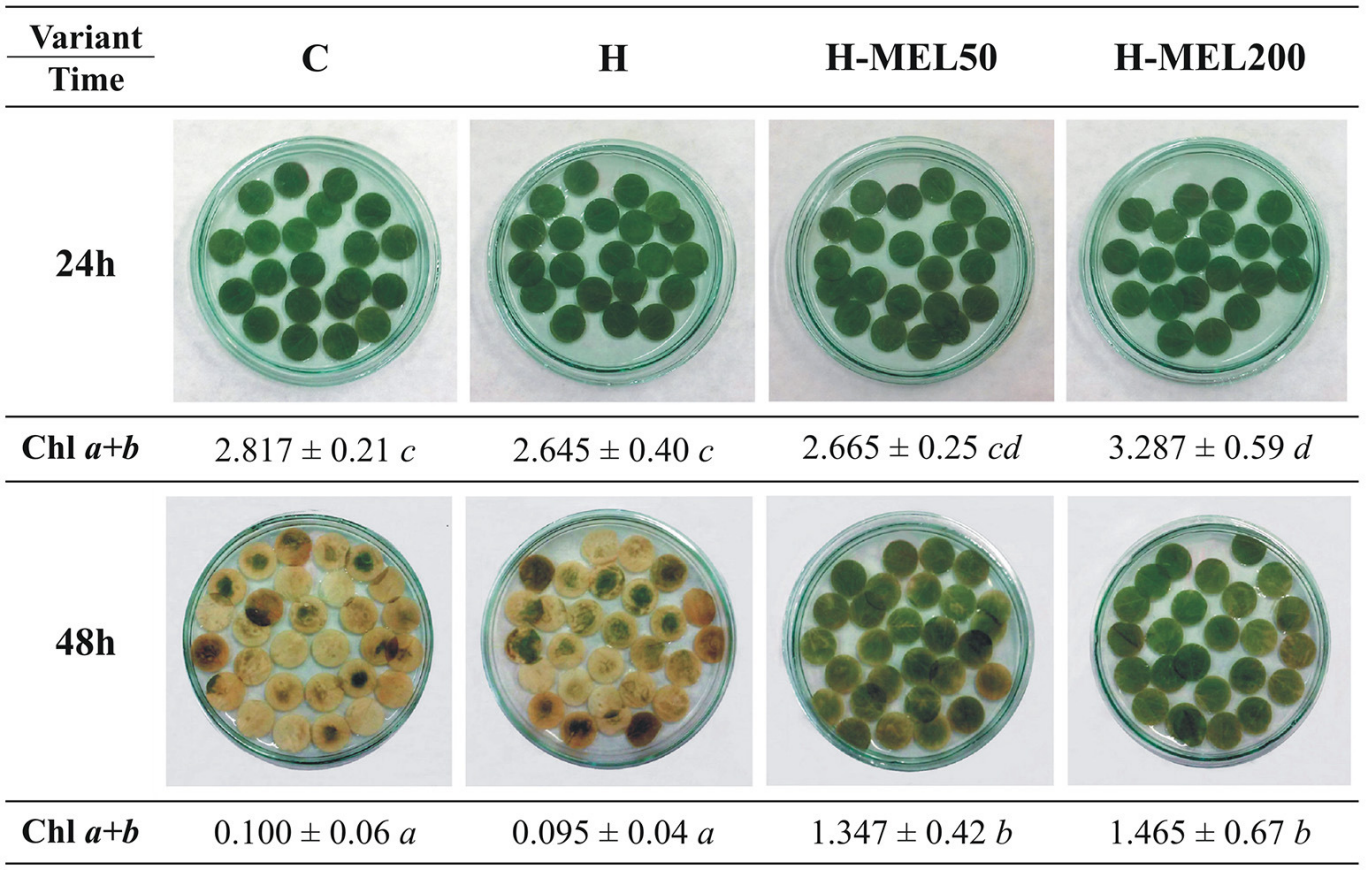
Changes in green color intensity and Chl *a*+*b* contents in leaf disks cut from 24-day-old pea plants grown from the control (C), hydroprimed (H), and hydroprimed with an aqueous solution of 50 μM (H-MEL50) or 200 μM melatonin (H-MEL200) seeds. Photographs were taken after 24 and 48 h of disks incubation in 75 μM PQ solution. As concerns Chl a+b contents [mg ${\text{g}}_{\text{FW}}^{-1}$]-two-way ANOVA and Duncan's *post-hoc* test were performed. The small letters in the table show statistical significance. ANOVA results: Chl a+b-Variant *F*_(3; 32)_ = 14.4 *p* < 0.0001; Time (24, 48 h) *F*_(1; 32)_ = 281.9 *p* < 0.0001; and interaction Variant × Time *F*_(3; 32)_ = 6.8 *p* < 0.001.

Next, we performed analyses on leaf disks incubated in 75 μM PQ for 24 or 48 h (Figure 4). After 24 h of PQ incubation there were no significant visual changes between the variants or in Chls *a*+*b* contents. 48 h PQ incubation triggered almost a 100% depigmentation of control and H leaf discs, while H-MEL50 and H-MEL200 preserved the green color and these disks contained only 50% less Chls *a*+*b* than those treated with PQ for 24 h (Figure 4).

**Figure 5:**
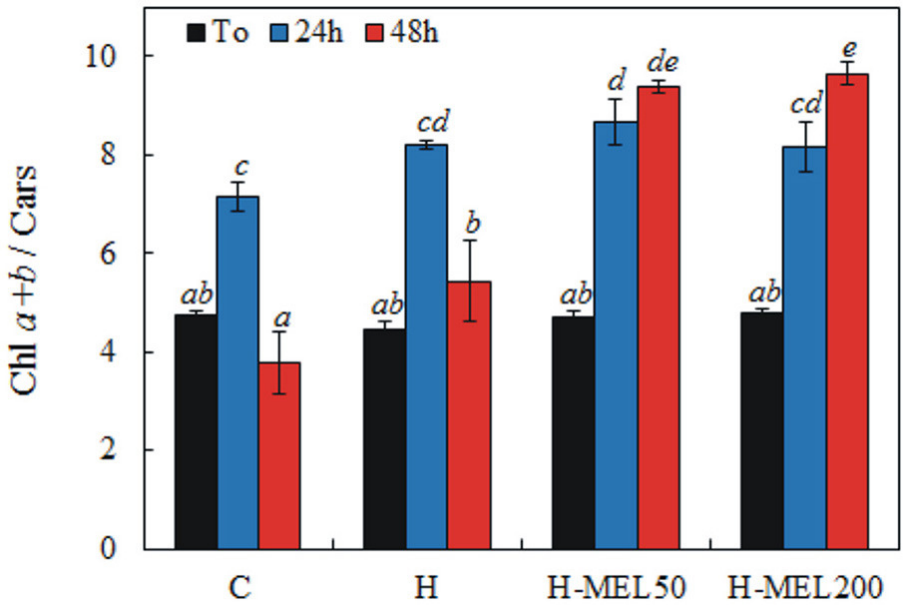
Ratio of chlorophyll *a*+*b* to carotenoids (Chl *a*+*b*/Cars) in leaf disks cut from 24-day-old pea plants grown from the control (C), hydroprimed (H), and hydroprimed with an aqueous solution of 50 μM (H-MEL50) or 200 μM melatonin (H-MEL200) seeds. Measurements were performed at T_0_ (before PQ treatment) and after 24 and 48 h of disk incubation in 75 μM PQ solution. The results are expressed as mean values of about 5 measurements ± SEM. Two-way ANOVA and Duncan's *post-hoc* test were performed. The small letters on the graphs show statistical significance. ANOVA results: Chl a+b/Cars-Variant *F*_(3; 44)_ = 26.6 *p* < 0.0001; Time (T_0_, 24, 48 h) *F*_(2; 44)_ = 85.9 *p* < 0.0001; and interaction Variant × Time *F*_(6; 44)_ = 15.4 *p* < 0.0001.

The ratio of Chl *a*+*b* and Cars before PQ treatment was almost the same in all investigated variants. 24 h of incubation in a PQ solution caused a significant increase in this ratio, which reached the highest value in H-MEL50 leaf disks. After 48 h of PQ treatment, Chl *a*+*b* and Cars ratio markedly decreased in control and H leaf disks, while in the variants with MEL the ratio continued to grow (Figure 5).

**Figure 6:**
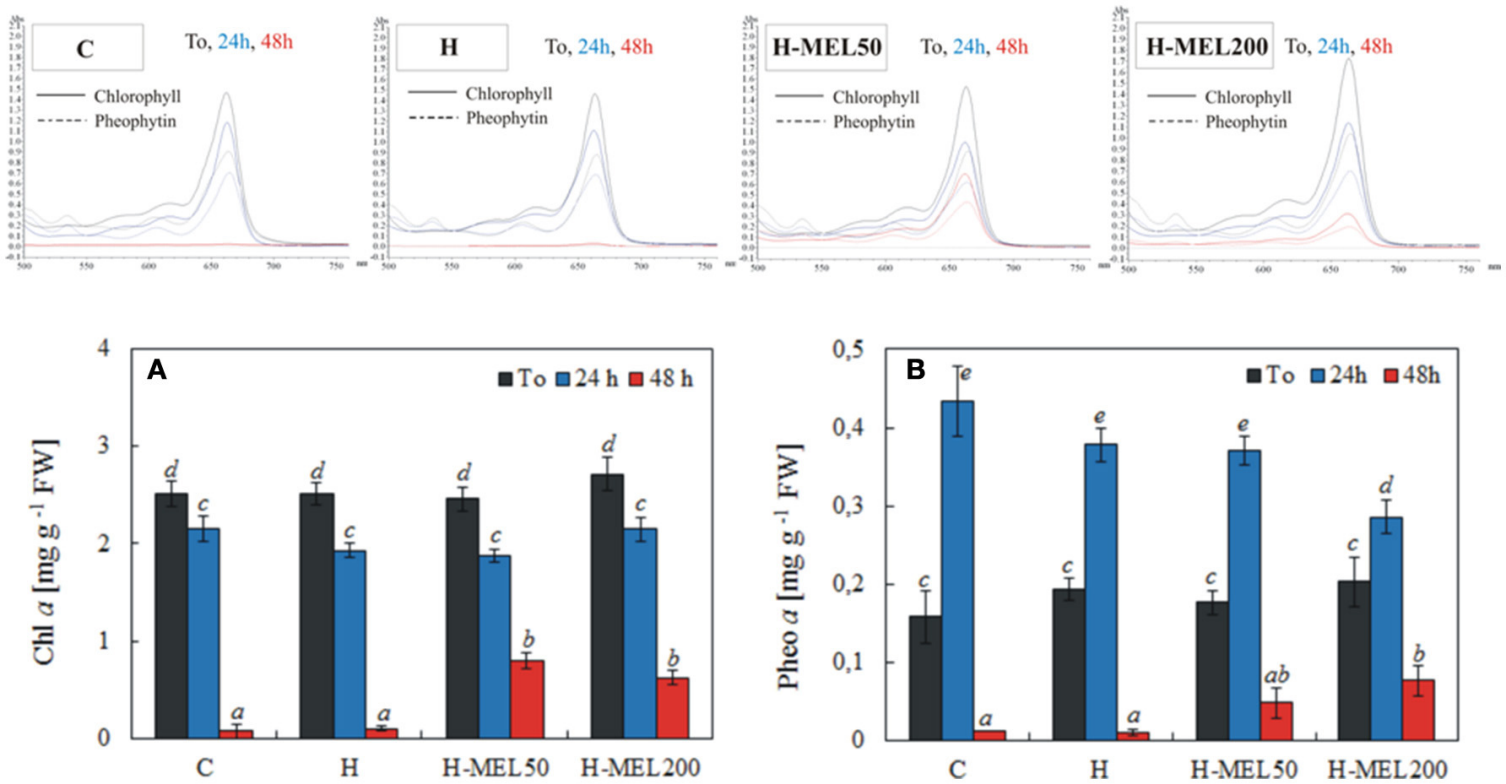
Chl *a* **(A)** and pheophytin *a* (Pheo *a*) **(B)** contents and scans of extracts from leaf disks cut from 24-day-old pea plants grown from the control (C), hydroprimed (H), and hydroprimed with an aqueous solution of 50 μM (H-MEL50) or 200 μM melatonin (H-MEL200) seeds. Measurements were performed at T_0_ (before PQ treatment) and after 24 and 48 h of disk incubation in 75 μM PQ solution. The results are expressed as mean values of about 5 measurements ± SEM. Two-way ANOVA and Duncan's *post-hoc* test were performed. The small letters on the graphs show statistical significance. ANOVA results: Chl a-Variant *F*_(3; 140)_ = 5.3 *p* < 0.001; Time (T_0_, 24, 48 h) *F*_(2; 140)_ = 443.8 *p* < 0.0001; and interaction Variant × Time *F*_(6; 140)_ = 4.8 *p* < 0.0001; Pheo a-Variant *F*_(3; 41)_ = 2.9 *p* < 0.1; Time (T_0_, 24, 48 h) *F*_(2; 41)_ = 154.9 *p* < 0.0001; and interaction Variant × Time *F*_(6; 41)_ = 3.8 *p* < 0.005.

Pheo *a*, a degradation product of Chl *a*, may interfere if it is present in the sample. The scans of Chl and Pheo and their quantitative analysis are presented in Figure 6. The level of Chl *a* slightly decreased after 24 h of PQ treatment in all investigated variants. Prolonged incubation time (48 h) caused a marked decline in Chl *a* content in control and H leaf disks, reaching only 3 and 4% of T_0_ values, respectively. In H-MEL50 and H-MEL200 leaf disks, this decline was not so drastic and Chl *a* levels achieved 33 and 23% of T_0_ values, respectively (Figure 6A). Before PQ treatment (T_0_) the lowest Pheo content was determined in control leaf disks, but after 24 h it increased almost 3 fold (Figure 6B). Although 24 h PQ-induced stress caused a significant rise in Pheo levels in all studied variants, the lowest accumulation was noticed in H-MEL200 leaf disks. With the rapid decline of Chl content after 48 h of PQ treatment (Figure 6A), in control and H leaf disks a trace amount of Pheo was also noticed, while in H-MEL50 and H-MEL200 leaf disks its content accounted for 27 and 47% of T_0_ values, respectively (Figure 6B).

**Figure 7:**
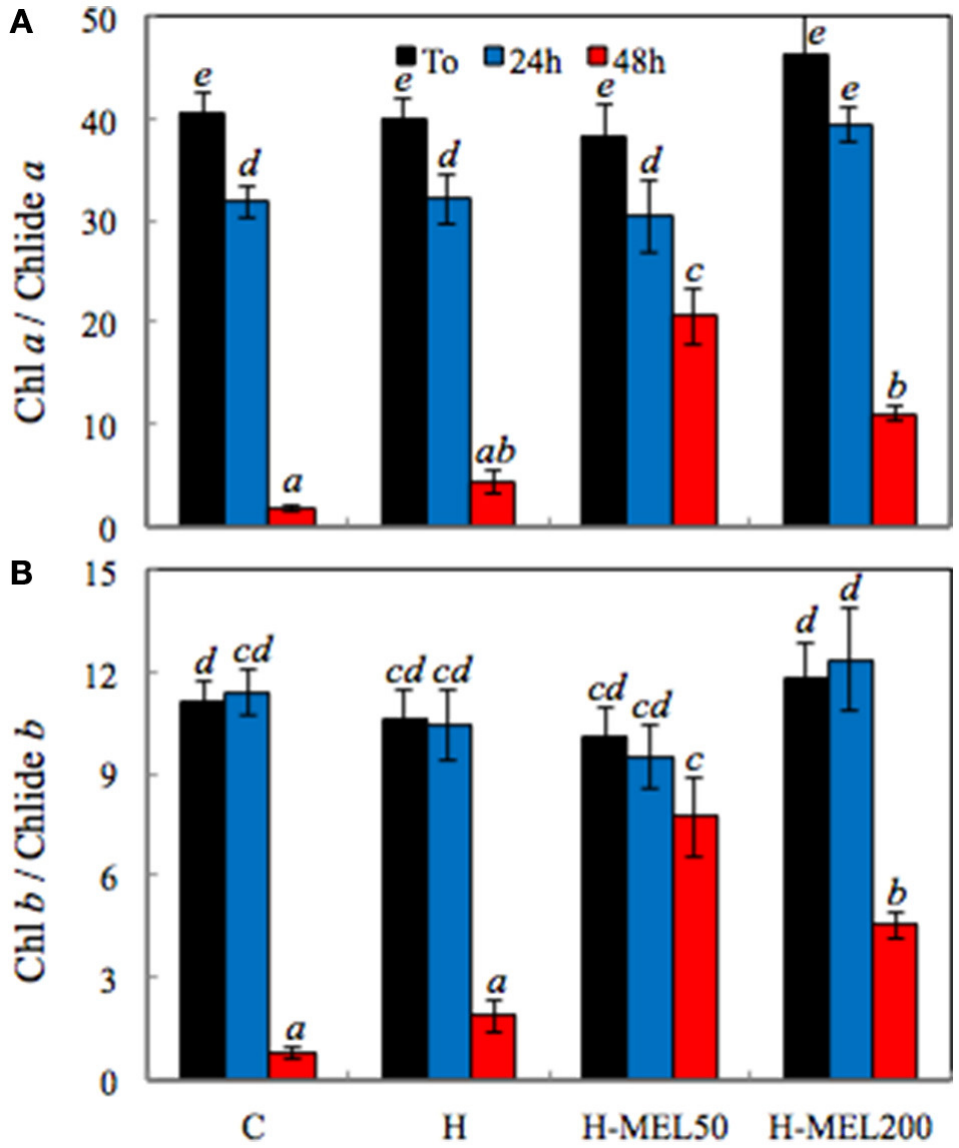
Ratio of chlorophyll *a*/chlorophyllide *a* (Chl *a*/Chlide *a*) **(A)** and chlorophyll *b*/chlorophyllide *b* (Chl *b*/Chlide *b*) **(B)** in leaf disks cut from 24-day-old pea plants grown from the control (C), hydroprimed (H), and hydroprimed with an aqueous solution of 50 μM (H-MEL50) or 200 μM melatonin (H-MEL200) seeds. Measurements were performed at T_0_ (before PQ treatment) and after 24 and 48 h of disk incubation in 75 μM PQ solution. The results are expressed as mean values of about 3 measurements ± SEM. Two-way ANOVA and Duncan's *post-hoc* test were performed. The small letters on the graphs show statistical significance. ANOVA results: Chl a/Chlide a-Variant *F*_(3; 24)_ = 7.0 *p* < 0.0001; Time (T_0_, 24, 48 h) *F*_(2; 24)_ = 169.8 *p* < 0.001; and interaction Variant × Time *F*_(6; 24)_ = 4.6 *p* < 0.001; Chl b**/**Chlide b-Variant *F*_(3; 23)_ = 3.9 *p* < 0.01; Time (T_0_, 24, 48 h) *F*_(2; 23)_ = 84.6 *p* < 0.0001; and interaction Variant × Time *F*_(6; 23)_ = 5.3 *p* < 0.001.

Chl decomposition is initiated with the separation of the phytol residue and the porphyrin ring of the Chl molecule, what results in Chlide formation (Kariola et al., 2005). The highest ratio of both Chl *a*/Chlide *a* and Chl *b*/Chlide *b* before PQ treatment was found in H-MEL200 leaf disks (Figures 7A,B). After 24 h of PQ-treatment Chl *a* / Chlide *a* ratio decreased about 20% in all investigated variants (Figure 7A), while Chl *b*/Chlide *b* remained at almost the same level (Figure 7B). Substantial changes appeared only after 48 h of PQ incubation, where both indicators sharply declined in control and H leaf disk, but in variants with MEL they remained high, especially after MEL50 treatment.

**Figure 8:**
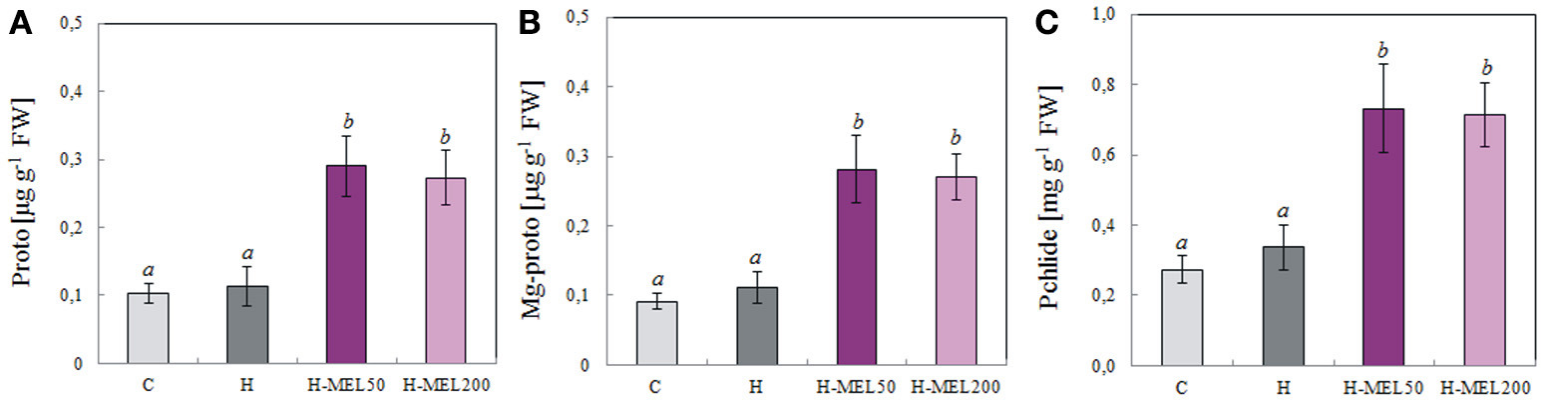
Contents of porphyrins: protoporphyrin (Proto) **(A)**, Mg-protoporphyrin (Mg-proto) **(B)** and protochlorophyllide (Pchlide) **(C)** in leaf disks cut from 24-day-old pea plants grown from the control (C), hydroprimed (H), and hydroprimed with an aqueous solution of 50 μM (H-MEL50) or 200 μM melatonin (H-MEL200) seeds. Measurements were performed after 48 h of disk incubation in 75 μM PQ solution. The results are expressed as mean values of about 8 measurements ± SEM. One-way ANOVA and Duncan's *post-hoc* test were performed. The small letters on the graphs show statistical significance. ANOVA results: Proto-Variant *F*_(3; 22)_ = 10.2 *p* < 0.001; Mg-proto-Variant *F*_(3; 22)_ = 9.7 *p* < 0.001; Philide-Variant *F*_(3; 23)_ = 9.8 *p* < 0.001.

To assess the relationship between porphyrin biosynthesis and exogenous MEL applied to the pea seeds, the effects of PQ-induced oxidative stress on the regulation of porphyrin biosynthetic intermediates in leaves were also examined. 48 h of PQ treatment caused a drastic decline in protoporphyrin (Proto), Mg-protoporphyrin (MgProto), and protochlorophyllide (Pchlide) in control and H leaf disks, while in H-MEL50 and H-MEL200 their levels were about twice as high. The content of all investigated porphyrins was slightly higher in the leaves of H-MEL50 variant (Figure 8).

## Discussion

Photosynthesis is a basic physiological process to maintain plant survival and stress factors disrupt this process. Research on the role of MEL in plants under different stress conditions has repeatedly demonstrated its positive impact on the functioning of the photosynthetic apparatus (Arnao and Hernandez–Ruiz, 2009; Sarropoulou et al., 2012; Wang et al., 2012, 2013a; Weeda et al., 2014; Liang et al., 2015; Szafrańska et al., 2016), but precise mechanism of MEL action still needs elucidation.

The first enzyme in Chl degradation is Chlase, so checking its activity in all examined leaf disks was necessary. In *A. thaliana*, MEL treatment inhibited Chlase (CLH1) gene expression (Weeda et al., 2014), but in our experiment activity of this enzyme was significantly higher in variants treated with MEL (Figure 3A). Chlase gene expression and *in vitro* activity in plant tissues sometimes do not correlate well with de-greening during physiological fruit ripening and senescence (Minguez-Mosquera and Gallardo-Guerrero, 1996; Fang et al., 1998), implying that either Chlase is not the rate-limiting enzyme in Chl break-down or that Chlase action is regulated post-translationally—what clarifies its latent function (Harpaz-Saad et al., 2007). This would explain some of the apparent discrepancies regarding the inhibition of CLH1 gene expression in *A. thaliana* treated with MEL, and the elevated activity of this enzyme in the analyzed pea leaves (H-MEL50 and H-MEL200). Because Chl levels are constantly modulated throughout the plant life and the catabolic pathway must be strictly controlled to avoid the excess of photodynamically active pigments, a post-translational regulatory mechanism is ergonomic and makes physiological sense; this allows the plant more flexibility (quick adaptation) in the response to environmental factors (Harpaz-Saad et al., 2007). It is also worth mentioning that transcriptome analysis of melatonin-treated *Arabidopsis* (Weeda et al., 2014) revealed upregulation of numerous genes implicated in the signaling of senescence-promotion but also stress-induced phytohormones, such as ethylene, abscisic, jasmonic, and salicylic acids (Jibran et al., 2013; Khan et al., 2014). This also might indicate that MEL provokes a stress response syndrome in plants as suggested by Kołodziejczyk et al. (2016a,b), although this issue still requires clarification. Since the breakdown of the damaged Chl and *de novo* synthesis are a natural processes occurring in plant cells, the next step was to examine if under oxidative stress MEL affects the contents of its precursor-ALA. Biosynthesis of ALA is the first step of Chl biosynthesis, and is therefore supposed to be a crucial control point in the regulation of Chl supply (Figure 2; Tanaka and Tanaka, 2006).

A wide variety of stresses cause a reduction in Chl biosynthesis due to downregulation of gene expression, decrease in protein abundance or post-translational modification of several enzymes participating in tetrapyrrole metabolism (Turan and Tripathy, 2015). In this work the question arose whether the positive effect of MEL on the function of the photosynthetic apparatus is associated rather with the accelerated synthesis of Chl and not with its delayed degradation. MEL applied into the pea seeds significantly enhanced ALA level immediately after PQ treatment of the leaves (Figure 3B), suggesting that MEL plays an important role in inducing *de novo* synthesis. In non-MEL treated plants (control and H) Chl biosynthesis pathway was downregulated by reduced ALA synthesis, as in sunflower leaves under salinity stress (Santos, 2004). Prolonged incubation (48 h) in 75 μM PQ medium triggered extensive discolouration of control and H leaf disks, while MEL at 50 or 200 μM alleviated PQ-induced photobleaching (Figure 4). This is consistent with the observations of Weeda et al. (2014), who noted that *A. thaliana* leaves treated with PQ in the absence of MEL became completely photobleached, while leaves treated with 1 mM MEL remained green.

Photosynthetic pigments including Chl *a*, Chl *b* and Cars are necessary for the photosynthetic process. The content of foliar pigments varies depending on species. Cars are thought to protect Chl from the absorption of excess energy which might otherwise photobleach the Chl. Variation in leaf pigments (Chls and Cars) and their proportion may be due to internal factors and environmental conditions (Sumanta et al., 2014). Their concentrations are closely related and the ratio of Chls *a*+*b* to total Cars is the greenness index of plants. This ratio generally decreases in senescing, unhealthy plants and rises in vigorously growing plants. In our experiment, before PQ-treatment (T_0_) there were no differences in Chls *a*+*b* and Car ratios between leaf variants, but after 24 h this ratio increased, reaching the maximum in H-MEL50 leaf disks (Figure 5). A significant decrease of this ratio in control and H leaf disks was observed after 48 h, while in variants with MEL (H-MEL50 or H-MEL200) it was still growing. This was associated with drastic decline of Chl and Car levels in control and H leaf disks and their much higher levels in variants due to MEL treatment (data not shown); this may confirm a positive influence of MEL on Chl and Car biosynthesis. Although in the present study a MEL effect on Car levels was shown, MEL did not appear to influence its concentration in shoot tip explants of the cherry rootstock PHL-C treated with different MEL solutions (Sarropoulou et al., 2012). These authors stated that there were no links between MEL concentrations and the Car biosynthesis pathway. However, a relationship between MEL concentrations and porphyrin contents was found, as in the present work. In H-MEL50 or H-MEL200 leaf disks, contents of all three investigated porphyrins (Proto, MgProto and Pchlide) were significantly higher than in control and H variants (Figure 8); this confirms that Chl biosynthesis is stimulated by MEL. This effect may depend on the concentration of MEL since as shown Rodriguez et al. (1994) and Kalka et al. (1997) in mammals, a high MEL concentration (1 mM) reduced the porphyrin content and decreased D-aminolevulinate synthase (ALA-S) activity. Because still little or no information for this aspect of the plant kingdom exist, further investigation are required to ascertain the precise role of MEL in Chl biosynthesis pathway.

Chlide has been long considered to be an intermediate of both Chl biosynthesis and breakdown (Takamiya et al., 2000; Hörtensteiner, 2006). However, recent studies have revealed that Chlide seems not to be a true intermediate of Chl catabolism and Chl degradation may process through formation of Pheo *a* as a Chl derivative (Schenk et al., 2007; Schelbert et al., 2009; Hu et al., 2013; Figure 1). According to Guyer et al. (2014) genes encoding highly conserved pheophytin-specific phytol hydrolase (PPH) are prevalent in higher plants, allowing the suggestion that Pheo-specific dephytylation by PPHs may be a common attribute of Chl decomposition during leaf senescence. Therefore, we checked the level of Pheo in all studied pea leaf disks following PQ treatment. After 24 h of PQ incubation, the Pheo level significantly increased in control leaf disks, while in H-MEL200 only a slight rise was observed (Figure 6B). These results may confirm a positive role of MEL in delaying Chl breakdown by formation of Pheo during PQ-induced oxidative stress. Supposing that in the first hours of PQ treatment Chlase activity increased in H-MEL50 or H-MEL200 leaf disks (Figure 3A), while after 48 h Chl/Chlide ratio was much higher in those variants (Figure 7), this may indicate that the long-term Chl breakdown involving Chlase was also limited by MEL.

The current findings suggest that under PQ-induced oxidative stress, MEL preserves the Chl content in pea leaves by delaying Chl breakdown and simultaneously accelerating its *de novo* synthesis. The current results are consistent with some other published findings. It is known that the activation of genes, or transcript measurements, do not always correlate with physiological effects because of post-transcriptional and post-translational regulation. In this work we documented such effects on the biochemical and physiological levels of several constituents.

## Author contributions

KS–work conception, all experiments concerning pea seeds and seedlings realization, data acquisition and analysis, drafting of the manuscript. RR–research consultation/discussion, manuscript revision: language and editorial corrections. MP–methodological consultant, statistical calculations, data analysis and interpretation, manuscript revision.

### Conflict of interest statement

The authors declare that the research was conducted in the absence of any commercial or financial relationships that could be construed as a potential conflict of interest. The reviewer TS declared a shared affiliation, though no other collaboration, with several of the authors MMP, KS to the handling Editor, who ensured that the process nevertheless met the standards of a fair and objective review.

## Abbreviations

AFMK: N^1^-acetyl-N^2^-formyl-5-methoxyknuramine
ALA: 5-aminolevulinic acid
ALA-S: D-aminolevulinate synthase
AMK: N-acetyl-5-methoxyknuramine
C-3HOM: cyclic 3-hydroxymelatonin
Cars: carotenoids
Chl: chlorophyll
Chlase: chlorophyllase
Chlide: chlorophyllide
CLH1: chlorophyllase gene
H: plants grown from hydro-primed seeds
MEL200: plants grown from seeds hydroprimed with 200 μM melatonin
MEL50: plants grown from seeds hydroprimed with 50 μM melatonin
HXK1: sugar-sensing and senescence associated hexokinase-1 gene
MEL: melatonin
MgProto: Mg-protoporphyrin
PAO: pheide *a* oxygenase
Pchlide: protochlorophyllide
Pheo: pheophytin
PPH: pheophytin-specific phytol hydrolase (pheophytinase)
PQ: paraquat
Proto: protoporphyrin
PS I: photosystem I
PS II: photosystem II
RNS: reactive nitrogen species
ROS: reactive oxygen species
SAG12: senescence-associated gene 12.
